# Protein Phosphatase 1ß Limits Ring Canal Constriction during *Drosophila* Germline Cyst Formation

**DOI:** 10.1371/journal.pone.0070502

**Published:** 2013-07-25

**Authors:** Shinya Yamamoto, Vafa Bayat, Hugo J. Bellen, Change Tan

**Affiliations:** 1 Program in Developmental Biology, Baylor College of Medicine, Houston, Texas, United States of America; 2 Department of Molecular and Human Genetics, Baylor College of Medicine, Houston, Texas, United States of America; 3 Jan and Dan Duncan Neurological Research Institute, Baylor College of Medicine and Texas Children’s Hospital, Houston, Texas, United States of America; 4 Medical Scientist Training Program, Baylor College of Medicine, Houston, Texas, United States of America; 5 Howard Hughes Medical Institute, Baylor College of Medicine, Houston, Texas, United States of America; 6 Division of Biological Sciences, Bond Life Sciences Center, University of Missouri, Columbia, Missouri, United States of America; National Cancer Institute, United States of America

## Abstract

Germline cyst formation is essential for the propagation of many organisms including humans and flies. The cytoplasm of germline cyst cells communicate with each other directly via large intercellular bridges called ring canals. Ring canals are often derived from arrested contractile rings during incomplete cytokinesis. However how ring canal formation, maintenance and growth are regulated remains unclear. To better understand this process, we carried out an unbiased genetic screen in *Drosophila melanogaster* germ cells and identified multiple alleles of *flapwing* (*flw*), a conserved serine/threonine-specific protein phosphatase. Flw had previously been reported to be unnecessary for early *D. melanogaster* oogenesis using a hypomorphic allele. We found that loss of Flw leads to over-constricted nascent ring canals and subsequently tiny mature ring canals, through which cytoplasmic transfer from nurse cells to the oocyte is impaired, resulting in small, non-functional eggs. Flw is expressed in germ cells undergoing incomplete cytokinesis, completely colocalized with the *Drosophila* myosin binding subunit of myosin phosphatase (DMYPT). This colocalization, together with genetic interaction studies, suggests that Flw functions together with DMYPT to negatively regulate myosin activity during ring canal formation. The identification of two subunits of the tripartite myosin phosphatase as the first two main players required for ring canal constriction indicates that tight regulation of myosin activity is essential for germline cyst formation and reproduction in *D. melanogaster* and probably other species as well.

## Introduction

The first step in sexual reproduction is the formation of functional male and female gametes. A key feature of gamete formation in many organisms is incomplete cytokinesis (IC), in which contractile rings during cytokinesis constrict, but do not fully close and generate cysts (groups of interconnected cells) [1]–[8]. The arrested contractile rings are then modified to form stable intercellular bridges, also known as ring canals, whose diameters increase at later stages of gametogenesis. Proteins, RNAs, and organelles are transported through these ring canals; thus, the primary function of IC is probably to ensure the efficient sharing of signals and resources between the connected cells.

**Figure 1:**
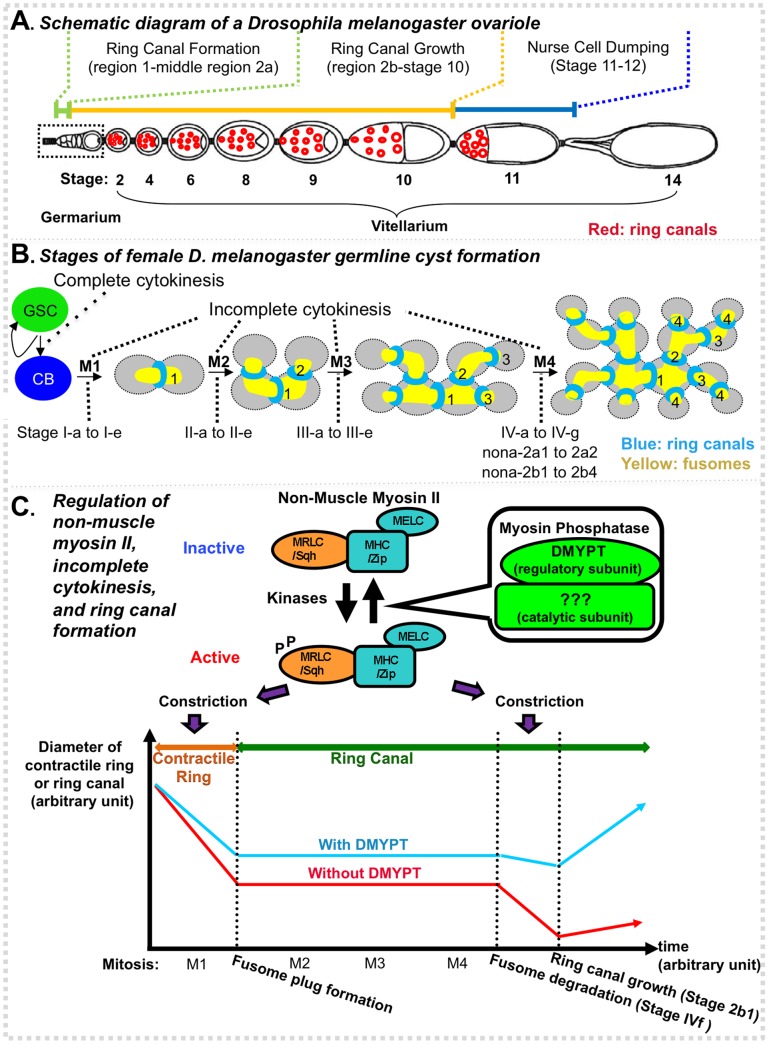
Germline cyst formation during *D. melanogaster* oogenesis. (**A**) A schematic drawing of an ovariole. An ovariole is composed of an anterior germarium (boxed) and a posterior vitellarium containing an array of developing egg chambers. The germarium is subdivided into regions 1, 2a, 2b and 3. The cyst in region 3 is also referred to as a stage 1 egg chamber. The vitellarium is subdivided into stages 2–14 with stage 14 being a mature egg. Ring canals start to grow in size in region 2b, reach their maximal sizes at stage 10, and degenerate after nurse cell dumping, a rapid phase of transporting the nurse cell cytoplasm into the oocyte. A schematic representation of some ring canals is shown in the vitellarium. (**B**) Stages of *D. melanogaster* germline cyst formation. *D. melanogaster* germ cells divide in a fixed pattern. Each mitotic division is characterized by five distinct stages (a-e). Following the final mitotic division, there are eight additional distinct stages in region 2 (IVf – nona-2b4) and a final one in region 3. Numbers beside ring canals (blue) indicate their mitotic origins. Note that ring canals are organized along the fusome (yellow). (**C**) Top: Regulation of non-muscle myosin II. Myosin II is a hexameric enzyme consisting of two heavy chains (MHC/Zip), two regulatory light chains (MRLC/Sqh), and two essential light chains (MELC) (only three subunits are shown here to simplify the image, the fly homologs are shown after "/"). The activity of myosin II is regulated by the phosphorylation of MRLC/Sqh. Myosin light chain kinase and several other kinases phosphorylate MRLC and activate myosin. In contrast, myosin phosphatase (MLCP) dephosphorylates phospho-MRLC and inactivates myosin. The myosin phosphatase is composed of three subunits, the myosin binding subunit MYPT (DMYPT in *D. melanogaster*), the catalytic subunit PP1cß (Flw in *D. melanogaster*), and a small subunit M20 of unknown function (no M20 has been identified in *D. melanogaster*). The DMYPT has been shown being required for IC, but whether Flw or M20 functions during IC is unclear. Bottom: A schematic view of incomplete cytokinesis and ring canal formation. An M1 ring canal is shown as an example. The same principle applies to M2, M3, and M4 ring canals but with different starting points. For each ring canal, its starting point is its birth mitotic division. The units for time and ring sizes are arbitrary. Neither the ring size nor the time is to scale. In wild-type flies, during each germline cystocyte mitotic division a contractile ring constricts and is then arrested when it reaches its maximal constriction point. A fusome plug forms in the arrested contractile ring and marks the conversion of the contractile ring into a ring canal. The fusome plug then fuses with the fusome from earlier mitotic divisions and grows to form a mature fusome. The ring canal does not change in size during the subsequent mitotic divisions. When all four mitotic divisions are finished, the fusome begins to degrade, and eventually disappears. Ring canals start to grow at stage nona-2b1, after a slight constriction. Similar events occur in *DMYPT* heterozygotes. In the homozygous *DMYPT* mutants, contractile rings constrict to a greater degree than those in heterozygotes resulting in smaller nascent ring canals. The ring canals remain at that size until the fusome starts to degrade. Although ring canals constrict only slightly after the final mitotic division in the presence of DMYPT, they constrict dramatically in its absence. Figure 1A are adapted and modified from [9], while 1B and 1C from [20].

We have recently shown that germline cyst formation in *D. melanogaster* females serves as a good model to study IC [9] (Figure 1A–B). In the germarium, a germline stem cell (GSC) divides asymmetrically via complete cytokinesis to form another GSC and a cystoblast (Figure 1B). The cystoblast then undergoes four-round mitotic divisions, via IC, forming a cyst with 16-interconnected cystocytes. Each IC proceeds through five distinct stages, with the four mitotic divisions being: (1) stages Ia to Ie, (2) IIa to IIe, (3) IIIa to IIIe, and (4) IVa to IVe. Then, the 16-cell cyst develops via nine additional stages, four in region 2a, four in region 2b, and one in region 3, resulting in a stage 1 egg chamber. The stage 1 egg chamber then leaves the germarium and continues to develop in the vitellarium through 13 stages, forming a mature stage 14 egg (Figure 1A). IC staging is based on the levels and distribution of anillin and α-spectrin immunostaining [9]. Anillin is a scaffolding cytokinesis protein that binds Actin and non-muscle myosin II (referred to as myosin II hereafter). Anillin localizes to the contractile ring, ring canal, and/or nuclei, with levels and distribution dependent on the cell cycle and the cyst age [9]–[15]. α-spectrin, an actin-crosslinking/scaffolding protein, localizes to membranous organelles called fusomes that are part of the continuous ER network, and is required for their formation [16]–[19].

In a previous study, we identified the *Drosophila Myosin Phosphatase Targeting Protein* (*DMYPT*) as a critical regulator of IC [20]. DMYPT is highly enriched in cells undergoing IC. Loss of *DMYPT* in germ cells results in over-constriction of contractile rings and ring canals during IC, especially after the fourth mitotic division and prior to ring canal growth (Figure 1C). As a consequence, minute ring canals form in *DMYPT* mutants that prevent intracellular nurse cell cytoplasm transport, resulting in small, non-functional eggs. *DMYPT* mutations have no effect on the number of mitotic divisions and do not affect cell fate determination of germ cells.

How *DMYPT* functions during IC is still unclear. Several studies have shown that MYPT can form a tripartite myosin light chain phosphatase (MLCP) with a catalytic serine/threonine Protein Phosphatase 1 (PP1) ß (also known as PP1δ in vertebrates) and a small subunit M20, and together the three inactivate myosin II by dephosphorylating phosphorylated myosin II regulatory light chain, encoded by the *spaghetti squash* (*sqh*) gene in *Drosophila* (Figure 1C) (reviewed in [21] and [22]). Thus, one hypothesis is that the *D. melanogaster* PP1ß, encoded by *flapwing* (*flw)* [23], functions during IC with DMYPT. However, Vereshchagina and colleagues found that *flw* played no role during early oogenesis, but instead was required for ring canal growth in late stages of oogenesis [24]. Recently, Sun and colleagues found that *flw* functions in follicle cells to control oocyte polarization, but they did not investigate the role of *flw* in the germ cells during IC [25]. *D. melanogaster* has three other PP1s, named according to their isotypes and cytological locations: PP1α87B, PP1α13C, and PP1α96A, as well as another DMYPT-like molecule, MYPT-75D [26], [27]. Flw binds both DMYPT and MYPT-75D, but unlike MYPT-75D, DMYPT also binds PP1α87B. Furthermore, vertebrate MYPT has been identified from cell culture studies as an interaction platform for a broad range of proteins [22]. A recent large-scale proteomic and interactomic study using Parallel Affinity Capture coupled to mass spectrometry found that Flw also has many binding partners [28]. Thus, whether other DMYPT-interacting proteins play regulatory roles during IC needs further investigation.

To identify additional players in IC, we performed an unbiased FLP/FRT-mediated germline mosaic screen on a collection of Ethyl Methane Sulfonate (EMS)-induced lethal mutations on the X chromosome [29]–[31]. Through screening of ∼1,800 X-linked recessive lethal stocks, we identified five mutations that show an IC phenotype, very similar to that seen in *DMYPT* mutants. The ring canals are severely over-constricted after the fourth mitotic division and before ring canal growth, although the constriction of contractile rings is arrested during the mitotic divisions of germ cells. Through genetic and molecular mapping of these mutants, all five mutations turned out to be alleles of *flw*. We found that Flw is expressed in germ cells undergoing incomplete cytokinesis, completely colocalized with DMYPT. Our genetic interaction data suggests that *flw* and *DMYPT* function together to negatively regulate myosin II activity. The discrepancy between this study and the earlier study by Vereshchagina *et al.* seems to have resulted from the difference in the allelic strengths used in the studies. The identification of two subunits of myosin phosphatase as the first two main players required to regulate ring canal constriction indicates that tight regulation of myosin activity is essential for germ cell development.

## Results

### A Germline Mosaic Screen of X-linked Essential Genes Identifies One Complementation Group, with Defects in Incomplete Cytokinesis Similar to *DMYPT* Mutants

To identify novel genes required for IC, we performed an unbiased genetic screen (Figure S1) of a collection of about 2,000 lethal mutations on the X-chromosome [29]–[31]. We reasoned that a mutation interfering with IC would cause oogenesis failure, leading to infertility, and the resultant ring canals would be small, as seen in *DMYPT* mutations, or be oversized, opposite to the phenotype seen in *DMYPT* mutants. Thus, we first screened for mutations laying no or only non-functional and deformed eggs under a dissecting microscope and then screened these oogenesis mutations for ones that affected ring canal morphogenesis with phalloidin staining analyses under a compound microscope. After that we investigated which mutants had IC defects by immunostaining and confocal microscopic analysis.

In the primary screen, we generated females carrying homozygous germline clones (GLCs) of the mutations using the Flippase-dominant female sterile (FLP-DFS) technique [40]. 1,798 of the crosses generated female offspring with the desired genotype. These mutant lines differed greatly in their ability to produce eggs (fecundity), with egg production ranging from 0 to over 40/animal/day. Eggs from some mutant lines also had highly aberrant morphology. Consistent with previous reports that most essential genes affect oogenesis [41], [42], we found that 1,073 mutations (60%) are associated with oogenesis defects with either diminished fecundity (laying less than 3 eggs/animal/day) or leading to eggs with morphological deformities.

**Figure 2:**
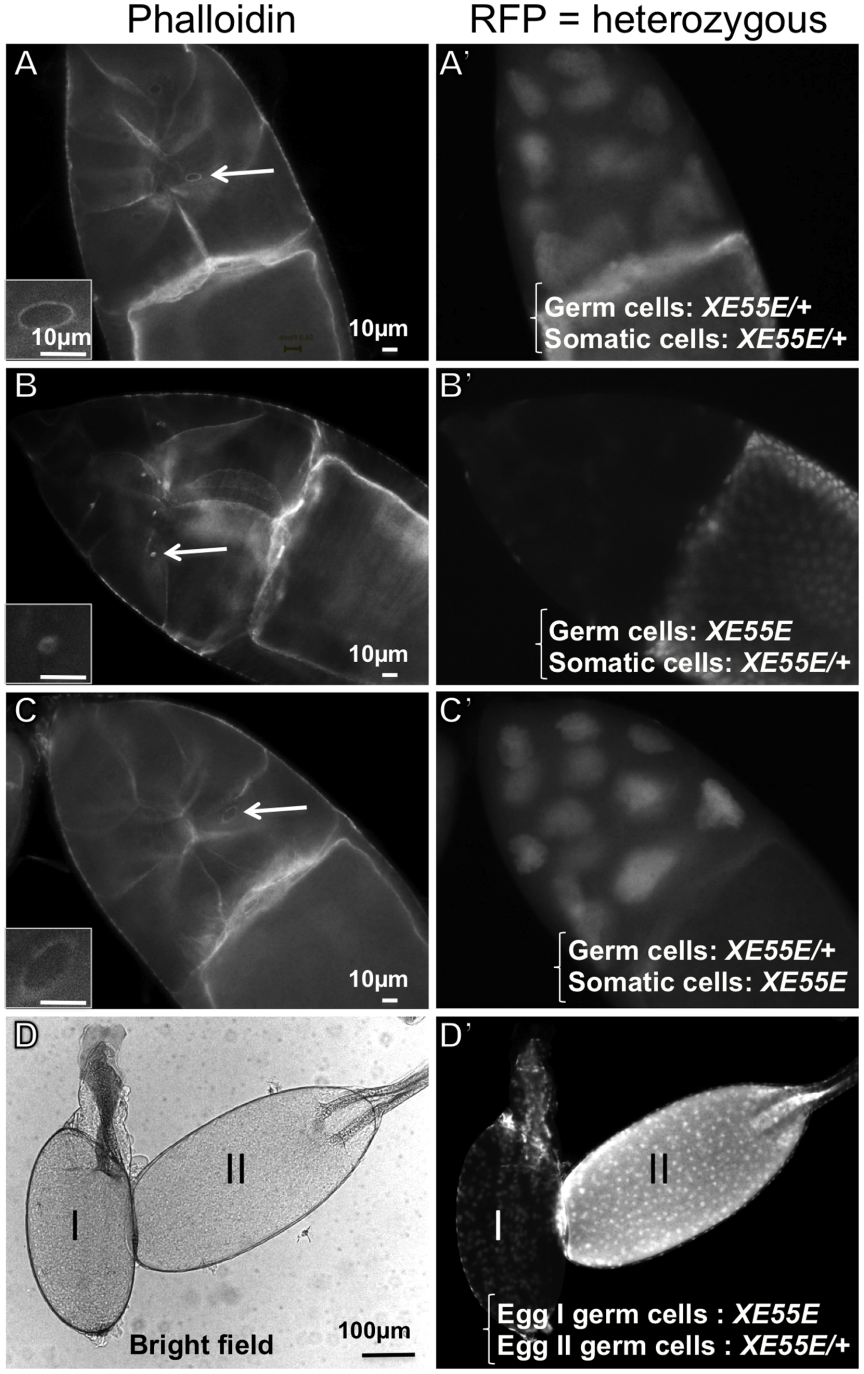
Homozygous XE55E germline clones, but not follicle clones, lead to formation of minute actin-ring canals and small eggs. (**A–C**) Stage 10 egg chambers with heterozygous *XE55E* (A), homozygous *XE55E* in germ cells (B), or homozygous *XE55E* in somatic follicle cells (C). Actin phalloidin-staining on the left and nuclear RFP images on the right. The genotypes of the germ cells and somatic cells of the egg chambers are as indicated in the figure. The heterozygous *XE55E* is *XE55E*/*P{ovoD1} y FRT19A hsflp*. The inserts are magnified views of the ring canals marked with arrows in each panel. Note that small ring canals formed only when the egg chamber contains homozygous *XE55E* in its germ cells. (**D**) Stage 14 eggs with homozygous *XE55E* (I) or heterozygous (II) *XE55E* in germ cells. White field image on the left and nuclear RFP image on the right. Scale bars: 10 µm for A–C, 100 µm for D.

In a secondary screen, we screened these 1,073 mutant lines with oogenesis defects for aberrant actin ring canal morphology. We generated flies carrying GLCs marked by the absence of red fluorescence protein (RFP) and stained their ovaries for F-actin with phalloidin. Five independent mutant lines exhibiting small actin ring canals throughout oogenesis were identified (Figure 2A-B and data not shown). No small ring canal phenotype was observed when the homozygous clones were only in the somatic follicle cells (Figure 2C), demonstrating that the gene is required in the germ cells to control ring canal morphogenesis. Homozygous GLCs of these mutants produced small, non-fertile eggs (Figure 2D, homozygous *XE55E* on the left and heterozygous on the right).

Interestingly, all 5 mutants failed to complement each other’s lethality, indicating that they are alleles of the same complementation group. We named this complementation group *XE55*, and refer to the alleles as *XE55A-E* in the order of identification. Being the first two mutations identified, *XE55A* and *XE55B* were used in most of the characterization experiments described later. The identification of only five mutations showing the same phenotype in a collection of about 2,000 mutations indicated that the screen was highly specific.

**Figure 3:**
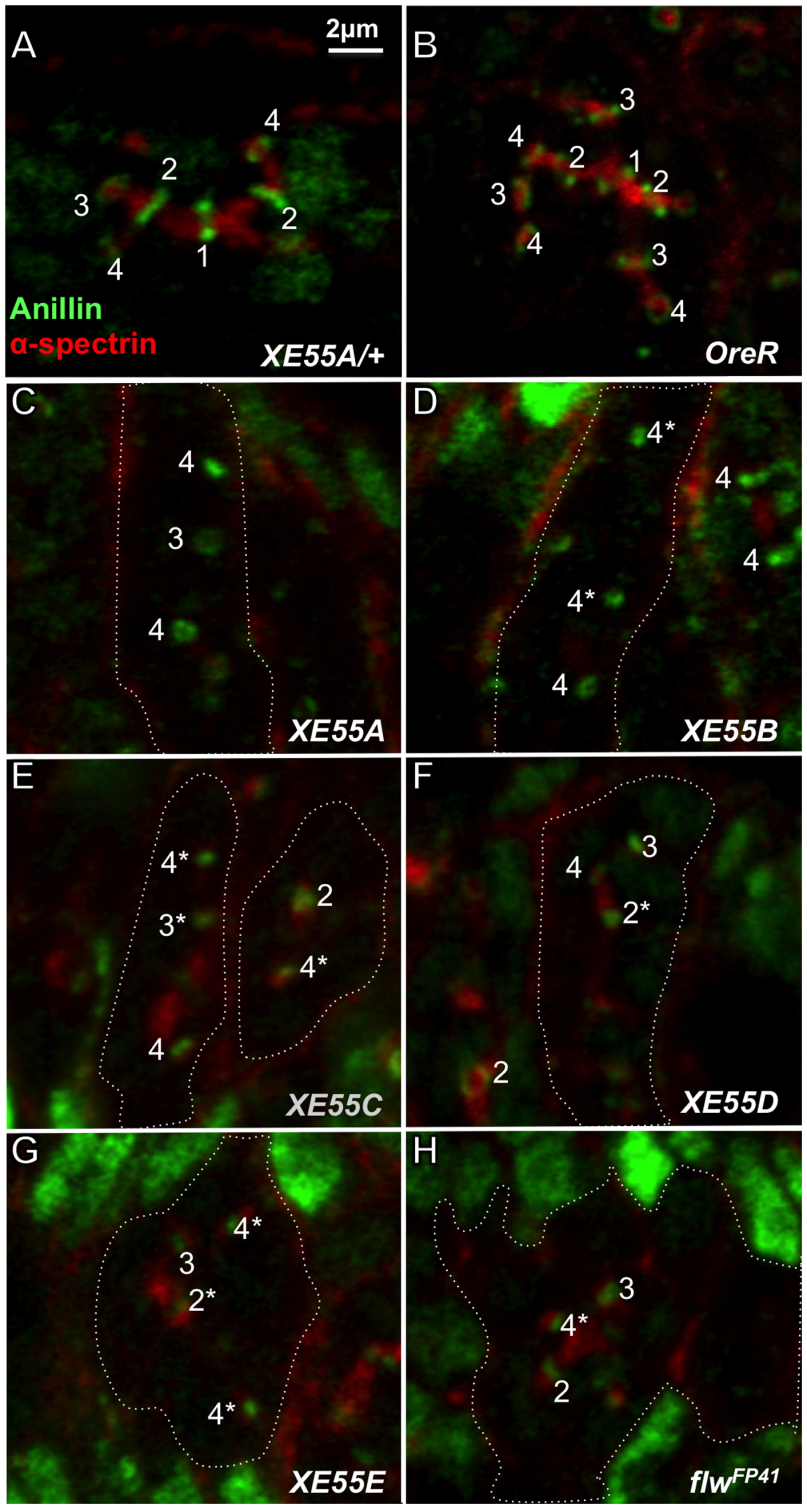
Homozygous GLCs of *XE55* mutations cause over-constriction of Anillin-stained ring canals during IC. Confocal images of part of germaria co-immunostained with antibodies against anillin (green) and α-spectrin (red). All cysts are at stage IVg, the sixth stage of the 4^th^ mitotic division. Homozygous germline clones are outlined with dashed lines. The mitotic origins of ring canals are labeled with 1, 2, 3, or 4 for the 1^st^, 2^nd^, 3^rd^, or 4^th^ mitotic division. The unmeasurable ring canals are marked with asterisks. (**A**) Heterozygous *XE55A*. (**B**) Wild-type (*OreR*). (**C–G**) Homozygous XE55s: *XE55A* (C), *XE55B* (D), *XE55C* (E), *XE55D* (F), or *XE55E* (G). (**H**) Homozygous *flw^FP41^*. Note that homozygous *XE55s* (C–G) and *flw^FP41^* (H) in germ cells caused formation of small ring canals. Two normal M4 ring canals from heterozygous *XE55B* (D, right) and one normal M2 ring canal from heterozygous *XE55D* (F, left) were also labeled for comparison. The genotypes for the heterozygous XE55s in this and all the following figures are *XE55^mutation^*/*ubi-RFP^NLS^ hsFlp^122^ FRT19A*. All panels have the same magnification. Scale bar: 2 µm.

To determine whether the small actin ring canals of GLCs of the *XE55* mutations result from a disruption of IC, we immunostained ovaries carrying RFP-marked GLCs with antibodies against Anillin, a marker for both contractile rings and early ring canals (Figure 3, green), and α-spectrin, a fusome marker (Figure 3, red). Ring canals in a heterozygous XE55 mutant (Figure 3A, and data not shown) are indistinguishable from those of wild-type (*OreR*) flies (Figure 3B, also compare movies S1 and S2). As expected, all *XE55* mutations disrupted IC, resulting in tiny Anillin-staining ring canals caused by over-constriction of contractile rings and ring canals (Figure 3C–G and movie S3). Some of the ring canals (marked with asterisks) were so small that no center void could be detected with light microscopy and classified as unmeasurable because their diameters were too small to be measured for quantitative analysis of the ring canal size for the following analyses. Such unmeasurable ring canals are extremely rare in wild-type cysts or in heterozygous *XE55 or* heterozygous *DMYPT* mutant cysts. The phenotype of the XE55 mutations is very similar to that of *DMYPT* mutants (Movies S1–3 and [20]). Surprisingly, we found that a strong lethal mutation of *flw* from Sun and colleagues [25] also produced the same IC defect (Figure 3H and Movie S4), contrary to the report by Vereshchagina and colleagues [24].

It is worth noting that in the *XE55* mutant GLCs, cystoblasts proceeded through the normal set of four mitotic divisions with IC to form 16 interconnected cells, with one differentiating into an oocyte (Figure 2B and data not shown). Fusome formation and distribution were also unaffected (Movies S1–3). Thus, these 5 mutations do not affect the number of mitotic divisions and the determination of cell fate and, thus, are all genuine IC mutations.

**Figure 4:**
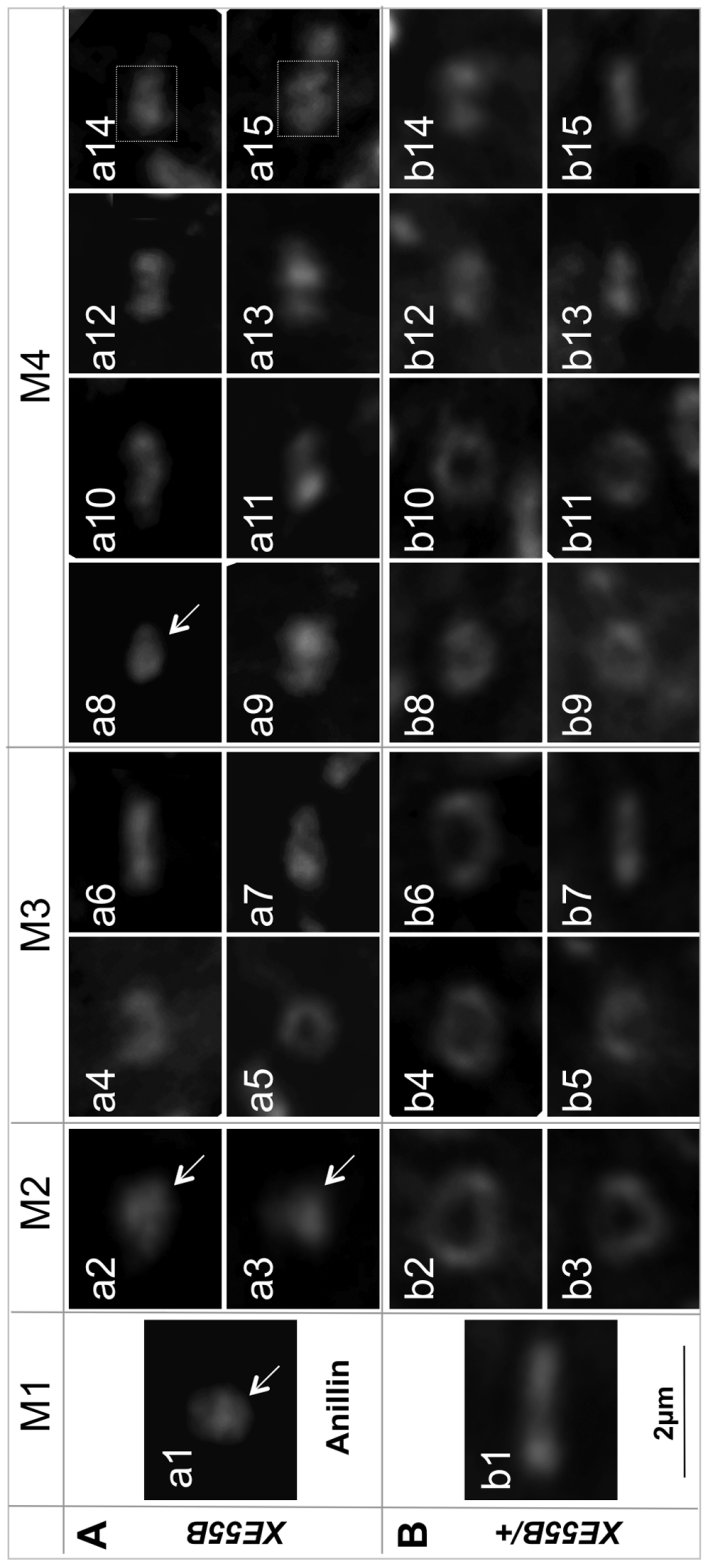
A comparison of the ring canals of stage IVg cysts carrying homozygous (A) or heterozygous (B) *XE55B*. The named ring canal of each panel is the one in the center of that panel (ring canals a14 and a15 were boxed to avoid confusion with surrounding ring canals). a1–15 are ring canals from a homozygous cyst, while the others belong to a heterozygous cyst. Mitotic origin of ring canals: M1 for a1 and b1; M2 for a2–3 and b2–3; M3 for a4–7 and b4–7; M4 for a8–15 and b8–15. Arrows point to unmeasurable ring canals. All panels have the same magnification. Scale bar: 2 µm.

To further characterize the precise defects occurring during IC in *XE55* mutant GLCs, we examined the 15 ring canals from a stage IVg homozygous *XE55A* cyst (Figure 4A) and compared the ring canal diameter to those of a same-age heterozygous cyst (Figure 4B). The ring canals in the homozygous *XE55A* cyst are obviously smaller; in fact, four of its 15 ring canals are unmeasurable (Figure 4A arrows).

**Figure 5:**
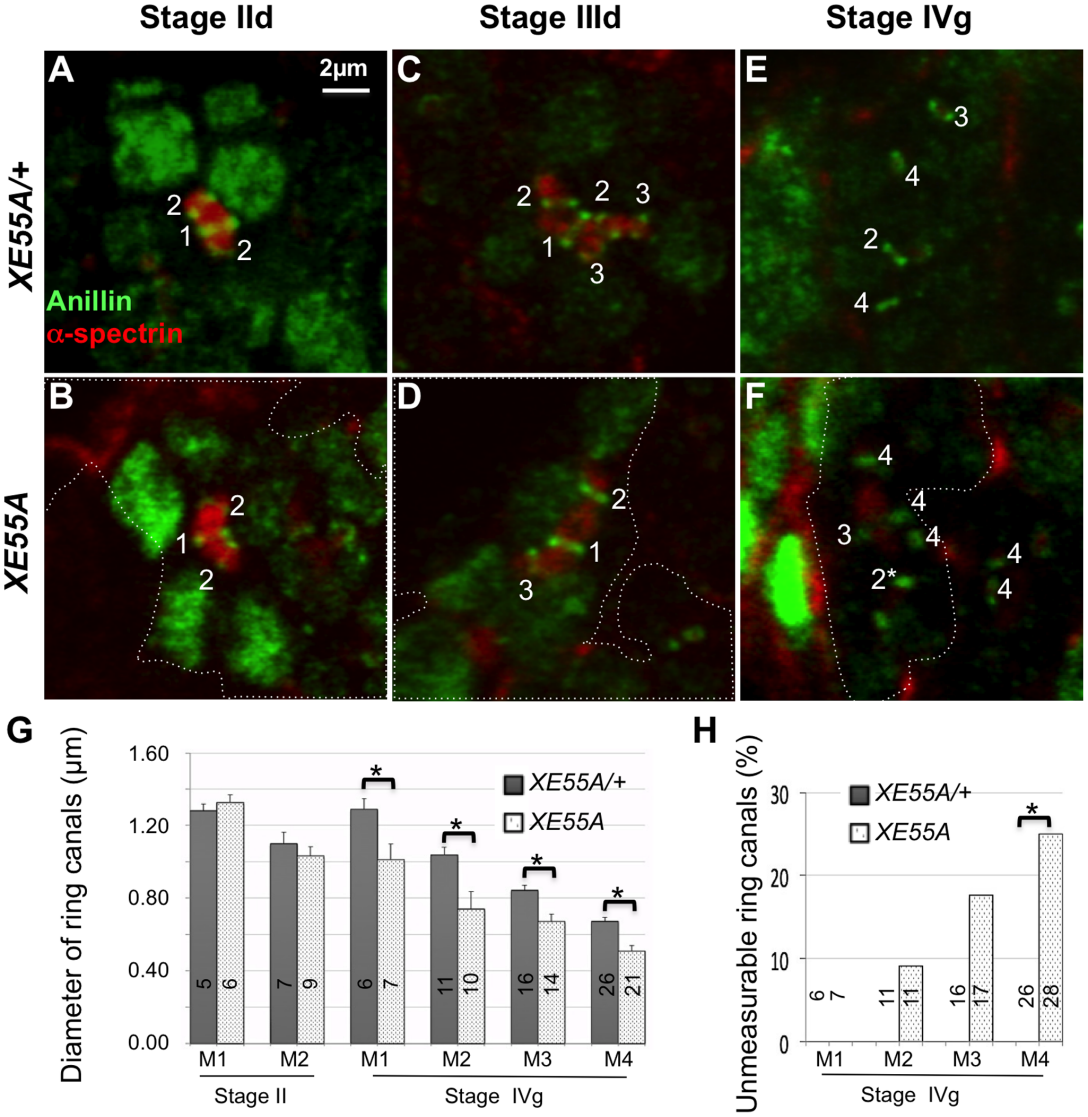
The *XE55* IC phenotype is more severe after all four mitotic divisions than during those divisions. (**A–F**) Confocal images of anillin (green) and α-spectrin (red) immunostained germline cysts at stages IId (A–B), IIId (C–D), and IVg (E–F). Genotypes of the germ cells housing the ring canals of interest are heterozygous *XE55A* for panels A, C, and E and homozygous *XE55A* for panels B, D, and F. Homozygous germline mosaic clones, marked by the absence of RFP (not shown), are outlined. The numbers mark the mitotic origins of ring canals. Two normal M4 ring canals from heterozygous *XE55A* (F, right) were also labeled for comparison; the top M4 ring canal appeared small because the current focal plane does not reveal its real diameter. All images have the same magnification. Scale bar: 2 µm. (**G**) Average diameters of ring canals of cysts with heterozygous (grey) or homozygous (doted) *XE55A*. Stage II is on the left and stage IVg on the right. (**H**) Percentage of ring canals too small to be measured. Error bars are standard error of the means. Numbers in panels G and H are ring canals measured or counted. The ring canal diameters that are significantly different (*P*<0.05) are marked with asterisks. 14 germaria were analyzed. Data shown is from a single representative experiment.

To determine at which stage the mutant phenotype becomes severe, we assessed the phenotypes of *XE55* mutant cysts at different stages. We found that *XE55* phenotypes were more severe after the four mitotic divisions were complete than during those divisions (Figure 5, Movies S1–3). For instance, at stages II or III, the ring canals of homozygous XE55A mutant clones (Figure 5B and D and movie S3) appeared similar to those of heterozygous (Figure 5A and C and movie S2) or wild-type (movie S1) cysts. However, at stage IVg, the sixth stage of the fourth mitotic division and before ring canal growth, the ring canals of homozygous *XE55A* mutant clones (Figure 5F and movie S3) were obviously smaller than those of heterozygous or wild-type cysts (Figure 5E and movies S1–2). This is in contrast to the behavior of a normal ring canal, which constricts only slightly after the fourth mitotic division, before they increase in size (movie S1 and [20]).

This observation was confirmed by a comparison of ring canal diameters of heterozygous and homozygous *XE55A* mutant cysts at two stages, stage II and stage IVg (Figure 5 G–H). Significant ring canal diameter differences were detected between heterozygous (grey) and homozygous (dotted) *XE55A* mutants at stage IVg but not at stage II (Figure 5G). Figure 5H shows the percentage of unmeasurable ring canals at stage IVg. These data show that the constriction of contractile rings in homozygote XE55 mutants were arrested, albeit constricted further than in heterozygote or wild-type flies, and that the newly formed ring canals do not change in size greatly while the cysts are actively dividing but over-shrink after mitotic divisions are finished, a phenotype reminiscent of *DMYPT* mutants (Figure 1C and [20]).

### *XE55* Complementation Group Maps to *flapwing*, Encoding the *Drosophila* Homolog of Protein Phosphatase 1ß

Through duplication mapping, involving flies carrying large X chromosomal segments translocated to the Y chromosome [43] and molecularly defined P[acman] BAC duplications [36], we mapped the lethality of XE55 mutations to a region containing the four genes, *flw*, *RabX2*, *CG12640*, and *CG32683* (Table 1 and Figure S2). Since *flw* encodes a predicted subunit of the myosin phosphatase, and since mutants for DMYPT, a regulatory subunit of myosin phosphatase, have similar IC defects [20], we hypothesized that *XE55* mutants are allelic to *flw,* even though this hypothesis was contrary to a previous report [24]. Indeed, *XE55* mutants failed to complement two previously reported semi-lethal *flw* mutations: *flw^6^*, an EMS-induced mutation, and *flw^7^*, a mutation caused by a *P*-element insertion [23], [24]. In addition, transheterozygotes for *XE55* and *flw^1^*, a viable, EMS-induced point mutation that when homozygous results in flightless flies [23], were also flightless. Furthermore, the lethality of *XE55* mutants (except *XE55C*) could be rescued by Gal4/UAS-induced expression of *flw* cDNA transgene in somatic cells (Table 1) [23], [24], [44]. Finally, an independently identified, strong *flw* mutation caused similar IC defect (Figure 3H above and movie S4). These data indicate that XE55 are alleles of *flw*, and thus we renamed *XE55A-E flw^XE55A−E^*.

**Table 1:** Lethal phases of *flw* alleles and rescue data.

		Rescue				
Allele Name	Lethal Phase	Duplication	UASt-Pp1ß9C			
		*Dp(1;3)DC224*	*tub-Gal4*	*maternal-Gal4*	*act-Gal4-II*	*act-Gal4-III*
**XE55A**	Pupa	Yes	No	NA	NA	NA
**XE55B**	Pupa	Yes	No	NA	NA	NA
**XE55C**	Pupa	Yes/no*	No	NA	NA	NA
**XE55D**	Pupa	Yes	No	No	Yes	Yes
**XE55E**	Larval	Yes	No	No	Yes	Yes
**flw^6^**	Pupa/Adult escapers	Yes	NA	NA	NA	NA
**flw^7^**	Pupa/Adult escapers	Yes	Yes	NA	NA	NA
**flw^FP41^**	Larval	Yes	No	NA	NA	NA

Note: Lethal phase is defined as the maximum stage the animals can reach. Some animals die at earlier stages, and if the culture is too crowded, the lethal phase can be earlier. The lethal phases of *flw^6^*, *flw^7^*, and *flw^FP41^* are listed as already published [23]-[25]. In addition, since the eggs for lethal phase analysis were from heterozygous females, maternal contributions were included. Maternal contributions cannot be avoided because Flw is required for oogenesis. All alleles except *XE55C* were rescued to fertile adults with an *flw*-carrying duplication *Dp(1;3)DC224*.

* : *XE55C* itself was not rescued, but *XE55C* females transheterozygous with *XE55D* or *XE55E* were rescued by *Dp(1;3)DC224* to fertile adults. This suggests that there is a second non-*flw* lethal hit on the *XE55C*-carrying chromosome.

For rescues with Pp1ß9C (*flw* cDNA under the control of UASt promoter, which expresses Flw-pB in somatic cells specified by Gal4 lines used [23], [24]), the following Gal4 lines were used: tub-Gal4 (*w; UAS-Pp1ß9C/CyO; TubGal4/TM6C*) expressing Gal4 under the control of the *α-tubulin84B* promoter, maternal Gal4 (w*; P{w[+mC] = matalpha4-GAL-VP16}V37) expressing GAL4-VP16 fusion protein under the control of the *α-tubulin67C* promoter, act Gal4-II (y^1^w*; P{w[+mC] = Act5C-GAL4}25FO1/CyO, y^+^) and act Gal4-III (y^1^w*; P{w[+mC] = Act5C-GAL4}25FO1/CyO, y^+^) expressing Gal4 under the control of *actin5C* promoter. For rescuing with tubGal4, males of *w; UAS-Pp1ß9C/CyO; TubGal4/TM6C* were crossed with females of *flw^mutations^/FM7*. For other Gal4-UASt-Pp1ß9C mediated rescues, females of *flw^mutations^/FM7; UASPp1ß9C/CyO* were crossed with males of corresponding Gal4 lines. For rescuing with duplication, males carrying *Dp(1;3)DC224* were crossed with females of *flw^mutations^/FM7*. For female rescues, rescued *XE55* mutant males were crossed with females of *flw^mutations^/FM7.* NA: not available.

**Figure 6:**
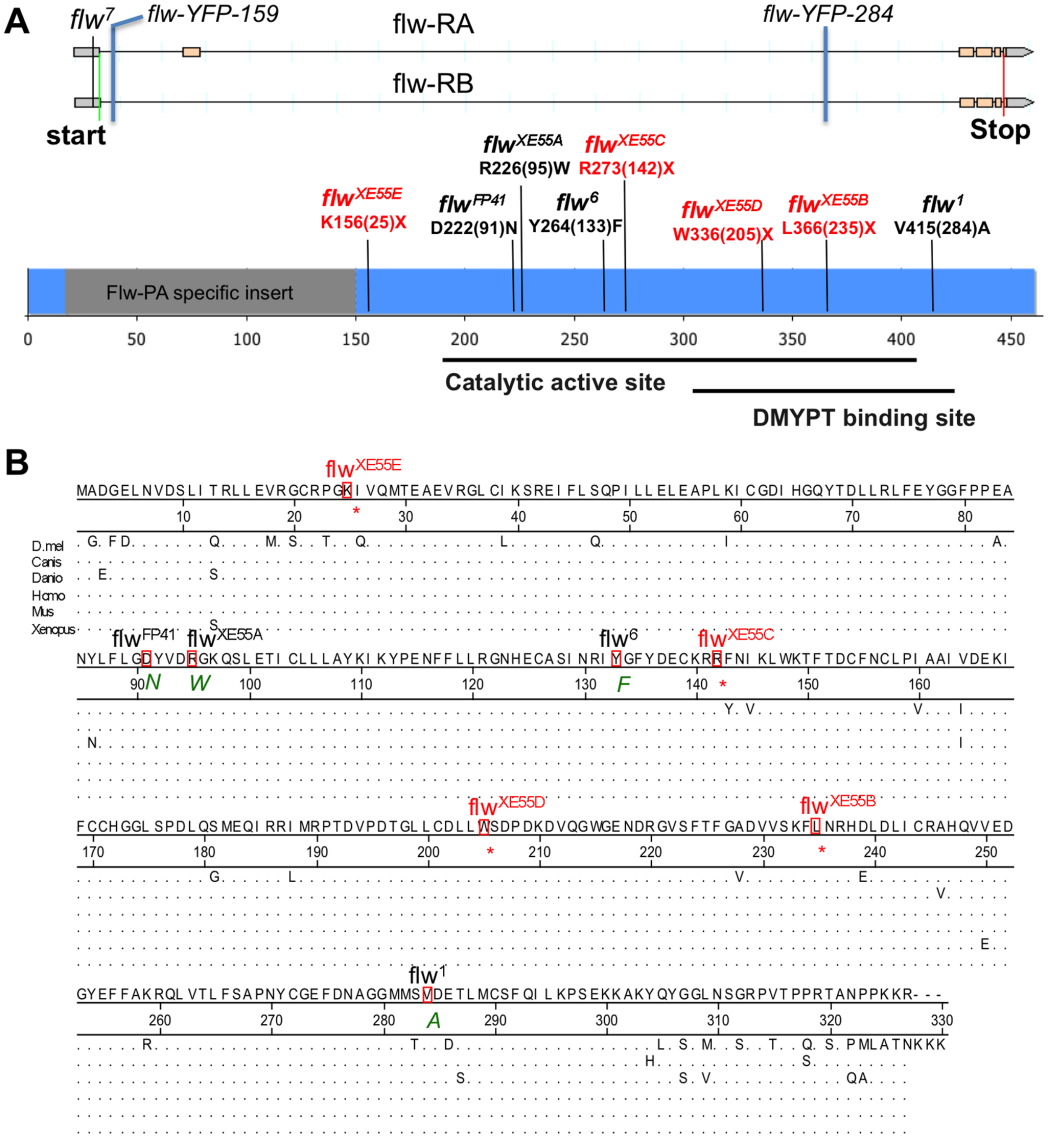
Mapping of *XE55* mutations. (**A**) Molecular nature of various *flw* mutations. Genomic annotation of *flw* is shown on the top, with the exons boxed. *flw^7^* is a *P*-element insertion in the 5′ untranslated region. *flw-YFP-159* and *flw-YFP-284* are two intronic *PiggyBac* yellow fluorescence protein trap lines. All other mutations are EMS-induced coding mutations. The three non-coding mutations are shown in the genomic map, while the coding mutations are shown in the annotated proteins. Protein regions shared between Flw-pA and Flw-pB are in blue. The Flw-pA-specific region is in grey. Amino acids are named with single letters. Positions according to the short isoform Flw-pB are in parentheses. Nonsense mutations are in red. *flw^1^*, *flw^6^*, *flw^7^*, *flw-YFP-159*, *flw-YFP-284*, and *flw^FP41^* have been described previously [23]–[25], [28]. Flw translation start and stop codons are indicated on the genomic map with green and red lines, respectively. The enzyme active site and the DMYPT binding site of Flw predictions are based on the Conserved Domain Database, which consists of a collection of well-annotated multiple sequence alignment models for full-length proteins (http://www.ncbi.nlm.nih.gov/Structure/cdd/cdd.shtml). (**B**) Flw mutations alter conserved residues. Protein sequence alignment of the short peptide pB of *D. melanogaster* Flw with PP1ß from Zebrafish (*Danio rerio)*, Frog (*Xenopus tropicalis*), Mouse (*Mus musculus*), Dog (*Canis lupus familiaris)* and Human (*Homo sapiens*). The amino acids mutated in Flw are boxed. Amino acid substitutes are included below each corresponding mutation, with “*”s indicate stop codons. Amino acids common to the majority of organisms are shown above the position lines and represented with dots in the alignment. Note that PP1ß is highly conserved across phylogeny, with only one amino acid difference between human PP1ß and *Xenopus* PP1ß or mouse PP1ß. The alignment was done using DNAStar software.

To identify the responsible molecular lesions, we isolated genomic DNA from hemizygous *flw^XE55A−E^* larvae and sequenced the *flw* open reading frame. *flw* is predicted to produce two transcripts through alternative splicing, a long (Flw-pA) and a short isoform (Flw-pB) [45]. The Flw-pA isoform has an extra, *Drosophila* genus-specific, coding exon compared to Flw-pB. As a consequence, Flw-pA is 131 amino acids longer than Flw-pB. We identified unique single point mutations in the *flw* coding region for each of the 5 alleles (Figure 6A). *flw^XE55A^* carries a missense mutation (Flw-pB R95W; Flw-pA R226W), while alleles *flw^XE55B−E^* carry various nonsense mutations. All *flw* mutations, including the five identified in our screens and those previously reported [23]–[25], [28] are shown in Figure 6. *flw^XE55A^*, *flw^FP41^*, and *flw^6^* may affect the catalytic active site of Flw, while *flw^1^* may affect the binding of Flw to DMYPT. We surmise that *flw^XE55E^, flw^XE55C^, flw^XE55D^* and *flw^XE55B^* may affect both the active site and DMYPT binding or lead to a protein with low stability due to the truncation. Since *flw^XE55E^* survives only to larval stages, while *flw^XE55A−D^* die at pupal stages (Table 1), *flw^XE55E^* may be a null allele or a strong hypomorph. Note that it was previously reported that *flw^FP41^* also die as larvae, while 1% of *flw^6^* and 2% of *flw^7^* hemizygous males survive to adult stages [23]–[25].

### Flw and DMYPT Colocalize in Cells Undergoing Incomplete Cytokinesis

**Figure 7:**
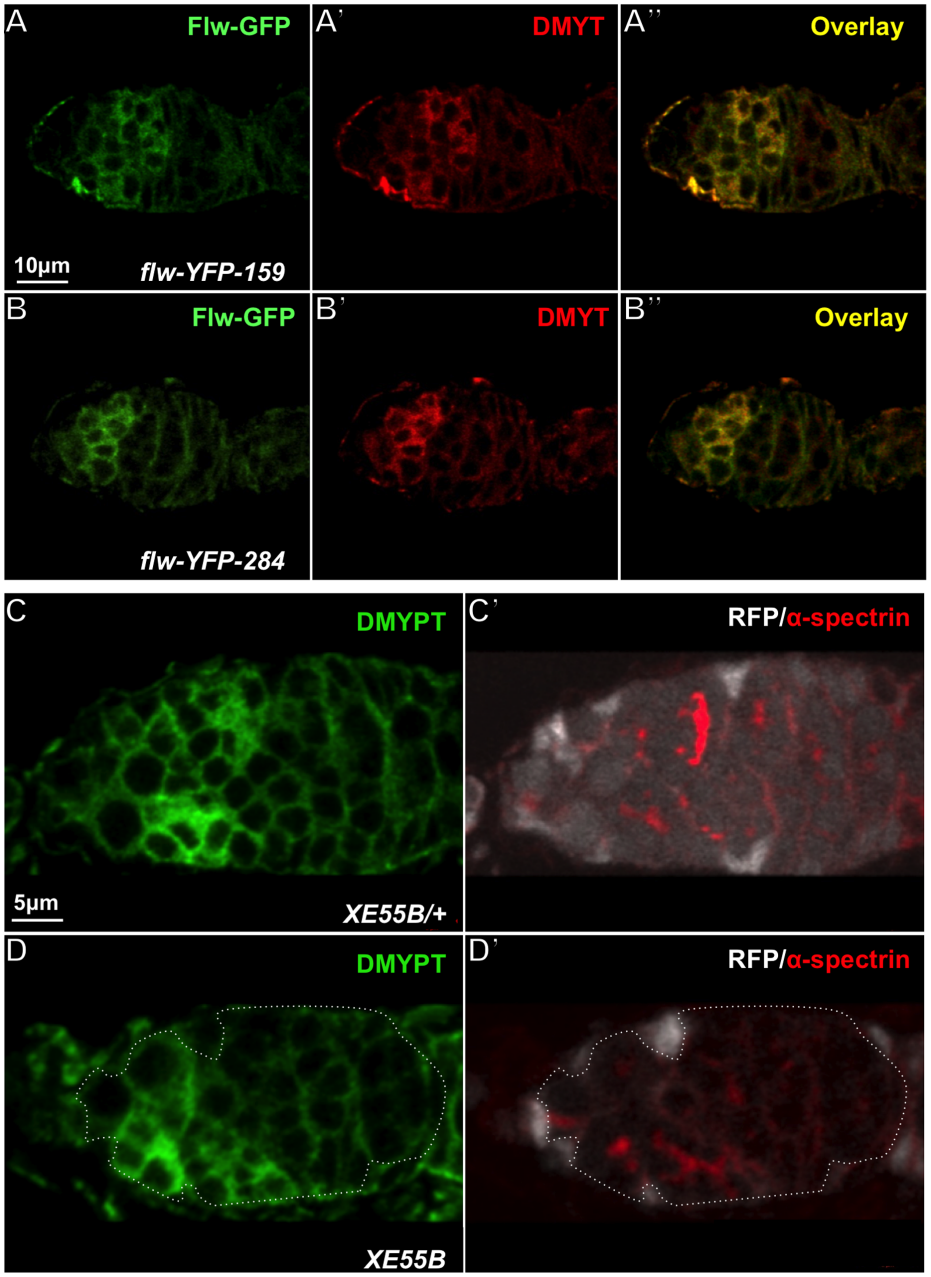
Flw is co-expressed with DMYPT in the germarium and *flw* mutations have no effect on the expression and localization of DMYPT. (**A–B**) Germaria of two independent Flw-YFP protein trap lines were stained with anti-GFP and DMYPT antibodies. Both Flw-YFP proteins colocalize with DMYPT signal in regions of the germaria where IC is taking place. (A) A germarium of *flw-YFP-159* line. (B) A germarium of *flw-YFP-284* line. (**C–D**) Germaria were immunostained with antibodies against DMYPT (green) and α-spectrin (red). Homozygous germline mosaic clones are marked by the absence of RFP (white). (C) A germarium with heterozygous *flw^XE55B^* mutation. (D) A germarium with homozygous *flw^XE55B^* germline clones. The boundaries of homozygous clones are outlined. A and B have the same magnification, so do C and D. Scale bars: 10 µm (A–B), 5 µm (C–D).

To determine whether Flw is expressed in the right place to play a role during IC, we obtained two independent Flw-yellow fluorescence protein (YFP) fusion protein-trap lines: *flw-YFP-159* and *flw-YFP-284* (Figure 6A and [28]). Both Flw-YFP fusion proteins were expressed in the germarium and enriched in the germ cells undergoing IC (Figure 7A–B). Since the expression pattern of Flw-YFP was very similar to that of DMYPT [20], we performed colocalization studies between Flw-YFP and DMYPT. Interestingly, the expression of both Flw-YFP proteins completely colocalized with DMYPT (Figure 7A–B, A’–B’, A”–B”). Therefore, Flw is localized in the right place to play its role during IC and it likely interacts with DMYPT in the process.

### Mutations in *flw* do not Affect DMYPT Expression and Localization During IC

Next, we wished to investigate whether DMYPT protein levels were altered in *flw* mutants. We found that DMYPT is enriched in the germ cells undergoing IC in a cyst stage-dependent manner but not in the germ cells undergoing complete cytokinesis or those that ceased dividing, regardless of the presence of *flw* mutants (Figure 7C–D and data not shown). Thus, *flw* mutants affect neither the expression levels nor the localization of DMYPT.

### Genetic Interactions between *flw*, *DMYPT*, and *myosin* During IC

**Figure 8:**
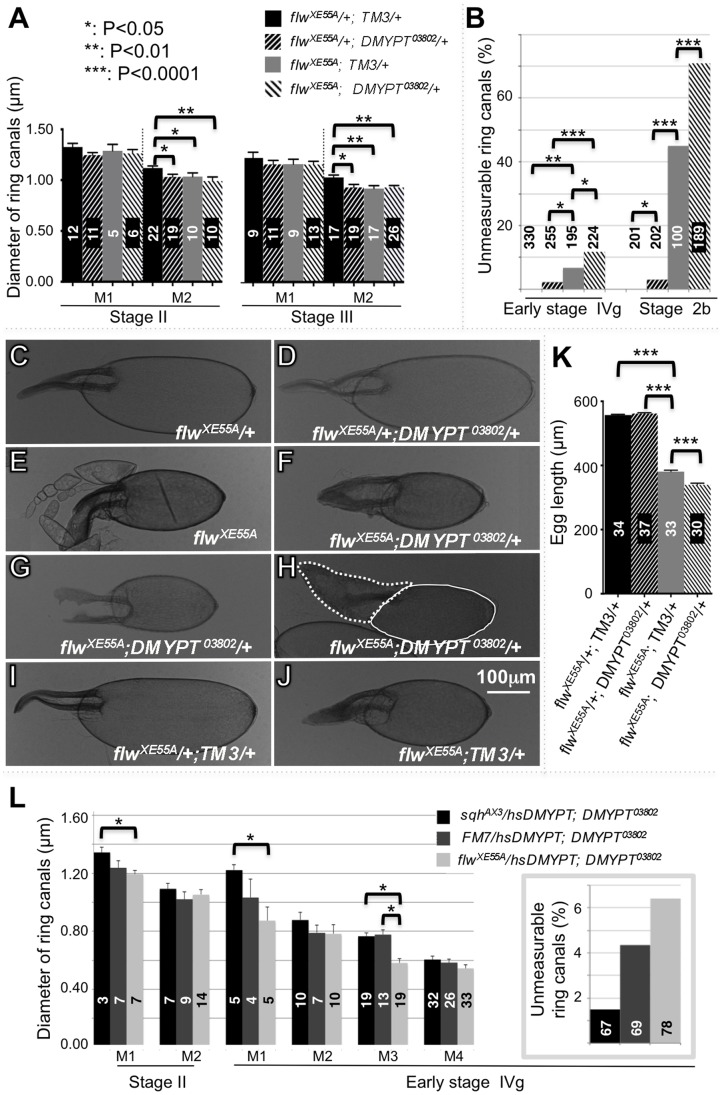
Genetic interaction of *flw*, *DMYPT*, and *sqh* during oogenesis. Data shown for each panel is from a single experiment, though all experiments were repeated. Numbers in the columns of the histograms are ring canals or eggs measured or counted. Error bars are standard error of the means. The values that are significantly different are marked with asterisks (*P<0.05, **P<0.01, ***P<0.0001). (**A**) Average diameters of ring canals of heterozygous *flw^XE55A^* with the balancer *TM3* (black) or a copy of *DMYPT^03802^* (upward slash), or homozygous *flw^XE55A^* germline clones with *TM3* (grey) or a copy of *DMYPT^03802^* (downward slash). Stage II is on the left and stage III on the right. 24 and 28 germaria were analyzed for those with TM3 and those without, respectively. (**B**) Percentage of unmeasurable ring canals at early stage IVg (left) and stage 2b (right) of the same germaria analyzed in panel A. (**C–J**) Stage 14 eggs developed from germline cysts with various levels of Flw and DMYPT: (C) Heterozygous *flw^XE55A^*, (D) Heterozygous *flw^XE55A^* with heterozygous *DMYPT^03802^*, (E) Homozygous *flw^XE55A^*, (F–H) Homozygous *flw^XE55A^* with heterozygous *DMYPT^03802^*, (I) Heterozygous *flw^XE55A^* with TM3, (J) Homozygous *flw^XE55A^* with *TM3*. All homozygous *flw^XE55A^* eggs were germline clones generated in the corresponding *flw^XE55A^* heterozygous females. All images have the same magnification. Scale bar: 100 µm. Eggs in panels F and H are slightly younger than those in other panels and still contain undegraded nurse cells. Nurse cells and the oocyte in panel H were outlined with dashed and solid lines, respectively. (**K**) Average length of mature eggs with various levels of Flw and DMYPT. (**L**) Average diameters of ring canals of homozygous *DMYPT^03802^*cysts with a copy of null mutation of *sqh* (*sqh^AX3^*, black), or the *FM7* balancer (dark grey), or a copy of *flw^XE55A^* (light grey). Stage II is on the left and early stage IV on the right. Insert: percentage of unmeasurable ring canals. Germaria analyzed: seven for sqhAX3-carrying flies, nine for FM7-carrying, and eight for *flw^XE55A^* -carrying.

Having observed that DMYPT colocalized with Flw but its protein levels were unaffected in *flw* mutants, we then explored whether *flw* and *DMYPT* genetically interact and are functionally related during IC. First, we generated homozygous *flw^XE55A^* germline clones in the presence or absence of one copy of the *DMYPT^03802^*, a *P*-element insertion-induced, hypomorphic mutation, and determined the ring canal sizes at two different stages, stages II and III (Figure 8A). Ring canal diameters or egg sizes that significantly differed are marked with asterisks in Figure 8 (*P<0.05, **P<0.01, ***P<0.0001). Note that the removal of one copy of *DMYPT* in the heterozygous *flw^XE55A^* background (upward slash) produced over-constricted ring canals to the same extent as the homozygous *flw^XE55A^* (grey) at both stages. Hence, *DMYPT* mutations enhanced the phenotype of a hypomorphic *flw* mutation and the two genes interact genetically. This conclusion was further supported by an increase in unmeasurable ring canals caused by halving DMYPT dosage (Figure 8B). Interestingly, the double heterozygous flies generated few (<3%) cysts with unmeasurable ring canals, and this did not change with cyst age (compare the percentage of unmeasurable ring canals at early stage IVg on the left of Figure 8B and stage 2b on the right). In contrast, unmeasurable ring canals increased with time in homozygous *flw^XE55A^* germline cysts (Figure 8B, compare the grey column at early stage IVg on the left with that at stage 2b on the right); by the time a homozygous *flw^XE55A^* cyst reaches region 2b, about half of its ring canals had become unmeasurable (Figure 8B right). Removing a copy of *DMYPT* significantly increased the frequency of unmeasurable ring canals at both stages and in heterozygous as well as homozygous flies (Figure 8B).

The consequences of the ring canal phenotypes are reflected in the eggs formed at the end. Heterozygous *flw^XE55A^* germline cysts produce normal eggs (Figure 8C and D). On the other hand, homozygous *flw^XE55A^* germline cysts formed small, non-functional eggs (Figure 8E), which were made even smaller by reducing *DMYPT* dosage (Figure 8F and G). These small eggs were likely the result of failure to transport nurse cell cytoplasm into the oocyte (Figure 8H). The presence of the TM3 balancer chromosome had no obvious effect on the phenotypes of the *flw^XE55A^* mutation (Figure 8, compare I with C and J with E). The morphological observations of the eggs were substantiated by a comparison of the egg length of flies with different levels of *flw^XE55A^* and *DMYPT* (Figure 8K).

Our comparisons of ring canal diameters of homozygous *DMYPT^03802^* cysts with one copy of *sqh^AX3^*, a null mutation of the myosin II regulatory light chain, or *flw^XE55A^* suggest that Sqh may be one of the targets of Flw during IC (Figure 8L). For ring canal size comparisons, we generated three kinds of cysts, all *DMYPT^03802^* homozygous (Figure 8L). Group I cysts (black) contained a copy of *sqh^AX^* ^3^ and thus should have the lowest level of active myosin II. Group II cysts (dark grey) contained an *FM7* balancer and should have a medium level of active myosin II. Group III cysts (light grey) contained a copy of *flw^XE55A^* so that they should have the highest level of active myosin II. Ring canals in Group I cysts were normally the largest, while those in Group III cysts were the smallest. While we didn’t observe a statistically significant (p<0.05) difference when DMYPT homozygous mutants alone were compared to DMYPT homozygous mutant in combination with *flw*/*sqh* alleles, we consistently observed a trend in which *flw* mutations enhanced, while *sqh* mutations suppressed *DMYPT* mutant phenotypes (Figure 8L). This conclusion is further supported by an increase or decrease in the number of unmeasurable ring canals from removing a copy of *DMYPT* or *sqh*, respectively (Figure 8L, insert). These genetic interactions suggest that Flw and DMYPT negatively regulate contractile ring constriction, while Sqh facilitates contractile ring constriction.

To determine whether phospho-Sqh (p-Sqh) levels were altered in *flw* mutant germaria, we performed immunostaining using several p-Sqh antibodies. However, the p-Sqh signal is very weak in the germline in both wild-type and *flw* mutant cells, while we observed that, in follicle cells, *flw* mutations had increased p-Sqh (Figure S3). Therefore, while the genetic interaction data suggests that Sqh is a potential target of Flw, the low endogenous p-Sqh levels in the germaria prevents us from conclusively stating that Sqh phosphorylation is altered in *flw* mutant cells during IC due to technical reasons.

## Discussion

Using an unbiased genetic screen of lethal mutants on the X chromosome, we have uncovered a second player mediating IC, *flw*, encoding the *Drosophila* homolog of serine/threonine Protein Phosphatase 1ß. We identified six independent alleles of *flw* that exhibits similar defects in IC, five from our screen and another from an independent forward genetic screen carried out by Sun *et al.* [25]. All six alleles had over-constricted ring canals similar to *DMYPT* mutants. Consequently, small ring canals were formed and the cytoplasmic transfer from nurse cells to oocyte was impaired, resulting in small nonfunctional eggs. Both colocalization and genetic interaction studies suggest that *flw* functions together with *DMYPT*, possibly to negatively regulate myosin activity of Sqh. Interestingly, the number of mitotic divisions and cell fates were unaffected, suggesting that *flw* is specifically required for regulation of IC.

### Myosin Phosphatase Functioning During IC

Our finding that *flw* functions during IC contrasts with the reported observation of Vereshchagina and colleagues [24]. While they found that ring canals in a *flw* mutant (*flw^6^*) are initially normal but failed to grow, leading to formation of small F-actin-staining ring canals at stage 10, we found that the ring canals of homozygous GLCs of *flw^XE55A−E^* are small throughout oogenesis. More importantly, we show that homozygous *flw^XE55A−E^* mutant GLCs show IC defects very early on, at a stage Vereshchagina and colleagues did not analyze in their studies. The discrepancy between their study and ours may be due to the different allelic strengths used, which is supported by our lethal phase analysis as well as the rescue results (Table 1). Furthermore, although we have not been able to directly investigate the effect of the *flw^6^* allele they used in their IC studies because the mutant stocks obtained from two sources have become viable and fertile (presumably due to the loss of *flw^6^* or the accumulation of suppressors), we found that GLCs of homozygous *flw^FP41^*, an independently isolated, strong allele [25], also produced over-constriction of contractile rings and ring canals, similar to XE55 alleles (Figure 3H and movie S4). The data that six different alleles of *flw* isolated independently from two laboratories caused IC defects strongly supports our conclusion that *flw* is indeed a gene necessary for successful IC. Since Flw and PP1ß are highly conserved, we speculate that PP1ß may function as a common factor mediating germline cyst formation in other organisms.

Several lines of evidence suggest that Flw and DMYPT form a *bona fide* myosin phosphatase during IC. Firstly, the mutations of *flw* and of *DMYPT* exhibited similar phenotypes (this study and [20]). Secondly, even though there are four PP1 encoding genes in *D. melanogaster*, the *flw* mutation alone is sufficient to cause a strong IC phenotype. This suggests that the role played by Flw during IC cannot be substituted by any other endogenous PP1s, either because they are not expressed in the germ cells undergoing IC or they function differently. Consistently, two of the four *D. melanogaster* PP1s, *PP1α13C* and *PP1α96A*, are not required for fertility [26], [27], and thus are unlikely to function during IC, at least not by themselves alone. The role of *PP1α87B* during oogenesis remains unknown, and we did not observe any germline clones with a null allele of *PP1α87B*, indicating that it may be essential for germline cell viability and have a more general housekeeping role (data not shown). Thirdly, mutations in *DMYPT* alone are sufficient to cause an IC defect [20], though another myosin-binding regulatory subunit, *MYPT-75D*, is present in the fly genome. DMYPT is most similar to human MYPT1, with both containing leucine zipper motifs at their C-termini, and highly conserved, inhibitory, Rho kinase phosphorylation sites in their central regions [46]. In contrast, *Drosophila* MYPT-75D is most similar to human MYPT3, lacking the Rho regulatory phosphorylation sites. Instead, MYPT-75D has SH3 sites and the C-terminal prenylation motif CAAX. Both MYPT-75D and DMYPT can bind p-Sqh and inactivate myosin II [24], [47]. Based on immunoprecipitation assays, DMYPT binds PP1α87B, as well as Flw, while MYPT-75D only binds Flw [24]. The physiological relevance of these interactions is unclear. No *MYPT-75D* mutants have yet been identified to date. Although we cannot rule out the possibility that *MYPT-75D* may also be involved in IC, it is unlikely that *DMYPT* and *MYPT-75D* have redundant functions in IC, as mutations in *DMYPT* alone are sufficient to cause IC defects [20]. Since the IC defects found in *flw* mutants are identical to *DMYPT* mutants, it is likely that Flw forms a complex with DMYPT during IC to negatively regulate myosin activity. This is supported by the colocalization of Flw and DMYPT in cells undergoing IC, as well as the enhancement of the *flw* mutation-caused oogenesis defects by *DMYPT* mutations and *vice versa*.

The involvement of *flw* and *DMYPT* during IC does not exclude their potential roles in later stages of oogenesis or in other tissues. In fact, both *flw* and *DMYPT* are known to be highly pleiotropic. For example, *flw* and/or *DMYPT* mutations in follicle cells affect oocyte polarity, egg chamber shape, and border cell migration and their mutations also interfere with other developmental processes [25], [37], [47]–[50]. Nonetheless, unlike homozygous *flw* mutations in germ cells, homozygous *flw* mutations in follicle cells do not affect ring canal morphogenesis (Figure 2C). Thus, Flw activity in germ cells is essential for IC and ring canal formation in a cell autonomous manner.

In conclusion, using an unbiased genetic screen, we have identified a novel role for *flw* during IC, unexpected from previous studies on the roles of *flw* during *D. melanogaster* oogenesis [24], [25]. Flw is expressed in cells undergoing incomplete cytokinesis, completely colocalized with DMYPT, and alleles of *flw* cause over-constricted ring canals during germline cyst formation. The identification of critical roles for both the catalytic subunit and the myosin binding subunit of myosin phosphatase during IC reinforces the importance of controlling myosin activity in this process.

## Materials and Methods

### Fly Strains

The fly strains we used include the following: *OreR*, *P{ovoD1} y FRT19A hsflp* from David Bilder (UC Berkeley [32], [33]), *flw^FP41^ FRT19A/FM7* from Trudi Schupbach (Princeton University [25]), *sqh^AX3^/FM7* from Roger Karess (Université Paris Diderot [34], [35]), *y w FRT19A*; *zip^1^/Cyo, Df(1)v-L15, y^1^/C(1)DX, y^1^ w^1^ f^1^; Dp(1;2)v^+^75d/+, flw^6^/FM7, flw^7^/FM7, ubi-RFP^NLS^ hsFlp^122^ FRT19A*, *DMYPT^03802^/TM3 Sb* and P[acman] BAC duplication lines [36] from the Bloomington *Drosophila* Stock Center (BDSC). *flw-YFP-159* (*w^1118^ PBac{681.P.FSVS-1} flw^CPT1001360^*) and *flw-YFP-284* (*w^1118^ PBac{681.P.FSVS-1} flw^CPT1002264^*) [28] were obtained from the *Drosophila* Genetic Resource Center (DGRC: Kyoto, Japan).

### Fly Husbandry and Crosses

Fly stocks were maintained on standard cornmeal-glucose food. Newly eclosed flies were fed with active yeast daily for optimal oogenesis until dissection. Germline clones (GLCs) were generated according to [37] using progeny of *y w mutant FRT19A/FM7c, Kr-GFP* crossed with *P{ovoD1} y FRT19A hsflp/Y* [32], [33] for the primary screen, and with *ubi-RFP^NLS^ hsFlp^122^ FRT19A/Y* for the remaining experiments.

### Lethal Phase Analysis

Eggs laid by *y w flw^XE55^ FRT19A/FM7c Kr-GFP* females were grown on standard grape juice-agar plates with yeast paste at room temperature. GFP-negative hemizygous male larvae were transferred onto new plates, and raised until they died. The stages the animals reached at the time of lethality were documented, and classified as “embryonic lethal”, “larval lethal”, “pupal lethal”, “semi-lethal (some survive to adulthood)” or “viable”.

### Genetic Interactions

To determine the genetic interactions between *flw* and *DMYPT*, we either fixed the copy number of *DMYPT* and altered the levels of *flw* or *sqh*, or fixed the copy number of *flw* and changed the levels of *DMYPT*. For the former, we crossed females of *sqh^AX3^/FM7; DMYPT^03802^ FRT2A/TM3* (or *y w flw^XE55A^ FRT19A/FM7; DMYPT^03802^ FRT2A/TM3*) with *hs-DMYPT; DMYPT^03802^/TM3* males and heat-shocked their offspring for 30 minutes in a 37°C water bath each day until eclosion. Flies carrying homozygous hypomorphic *DMYPT^03802^* mutation were generated by rescuing the mutants with heat-shock induced ectopic expression of *DMYPT* (*hsDMYPT*). After a four-day depletion of the heat-shock-induced DMYPT, homozygous *DMYPT^03802^* flies were dissected and their ovaries immunostained. For the latter, *y w flw^XE55A^ FRT19A/FM7, Kr-GFP* were crossed with *ubi-RFP^NLS^ hsFlp^122^ FRT19A/Y; DMYPT^03802^/TM3*. Homozygous germline clones were induced by heat-shocking their offspring at 37°C at the third instar larval stage for two consecutive days, two hours each day. Two days after eclosion, females were placed into yeasted vials for two days with *FM7* males, and then dissected.

### Immunohistochemistry

Immunostaining was performed according to [20]. F-actin staining with fluorescently labeled Phalloidin was performed as in [37]. The following primary antibodies were used: rabbit anti-DMYPT antibody, preadsorbed with fly embryos, 1∶100 [20], rabbit anti-Anillin, 1 µg/ml (a gift from Christine Field [10]), mouse anti-α-spectrin 1∶100 (Developmental Studies Hybridoma Bank (DSHB) [38]), mouse anti-GFP (1∶500) (Invitrogen, mAb 11E5 and mAb 3E6), and several antibodies for phosphorylated Sqh: rabbit anti-phospho-MLC2 S19 1:250 and rabbit anti-phospho-MLC2 T18S19 1:250 from Cell Signaling Technology, rabbit anti-phospho-Sqh 1∶400 from Luke Alphey [24], guinea pig anti-Sqh 1P 1∶500 and rat anti-Sqh 2P 1∶3000 from Robert Ward [39]. Secondary antibodies (Invitrogen) used were Alexa488, Alexa546, or Alexa647 anti-rabbit, mouse, rat, or guinea pig (highly cross-adsorbed if available) (1∶500).

Images in Figure 2 and Figure 8C–J were taken using a Leica DM5000 microscope. All others were captured using a Zeiss LSM 510 META NLO (63X oil C-Apochromat objective, zoom 2x, Z step size 0.5 µm) and analyzed using the LSM Image Browser. All figures were prepared using PowerPoint (Microsoft) and Photoshop (Adobe).

### Statistics

Statistics were performed using GraphPad Prism v. 6 for Mac (GraphPad Software, La Jolla, CA, USA): Figure 5G, as well as Figure 8 A, K, and L: multiple comparisons using Ordinary one-way ANOVA nonparametric tests, Figure 5H, Figure 8B, and the insert of Figure 8L: pair-wise comparisons using two-sided Fisher’s exact tests.

## Supporting Information

Figure S1 **A flowchart of the procedures to isolate mutations causing defective incomplete cytokinesis.** IC mutations were identified in four steps: screening the 1,798 lethal lines for mutations disrupting oogenesis, screening the 1,073 oogenesis mutations for those affecting actin ring canal morphogenesis, screening the small ring canal mutations for IC mutations, and mapping of the IC mutations.
(TIF)

Figure S2 **Mapping of XE55 mutations.** (**A**) Duplication mapping of XE55s between X chromosome bands 9B1 and 9F2. Duplications were shown as boxes below a drawing (by C.B. *Bridges*) of part of a polytene X chromosome. The duplication that rescued the mutation is labeled in pink, while those did not rescue are in grey. Same is true for panel B. (**B**) Refinement of the locations of the mutations with P[acman] BAC duplications to a region containing four genes. Above the duplications is a gene annotation of the relevant chromosomal region (adapted from http://flybase.org).
(TIF)

Figure S3 **Mutations of** ***flw*** **in follicle cells caused an increase of phosphorylated Sqh.** Immunostaining of a stage 10 egg chamber from a *XE55A*/*ubi-RFP^NLS^ hsFlp^122^ FRT19A* fly with a phospho-myosin light chain 2 (Ser19) antibody (Cell Signaling Technology, Inc. #3671), which recognizes Sqh phosphorylated at Ser21 (red). Panels A and B are two different focal planes of the same egg chamber. Homozygous clones (boxed with dashed lines) were marked by the absence of RFP (white). Note the increase of phosphorylated Sqh in all the clones. Scale bar: 10 µm.
(TIF)

Movie S1 A confocal Z-stack of a germarium from an *OreR* fly immunostained with antibodies against anillin (green) and α-spectrin (red).
(ZIP)

Movie S2 A confocal Z-stack of a germarium carrying heterozygous XE55A mutant. Green: anillin, red: fusome, white: RFP. Note that the heterozygous cysts are similar to the wild type cysts shown in Movie 1.
(ZIP)

Movie S3 A confocal Z-stack of a germarium carrying homozygous XE55A mutant clones. Green: anillin, red: fusome, white: RFP. Homozygous clones are RFP negative. Note that the homozygous clones are similar to the heterozygous cysts in region 1 but contain abnormally small ring canals in region 2.
(ZIP)

Movie S4 A confocal Z-stack of a germarium carrying homozygous *flw^FP41^* mutant clones. Green: anillin, red: fusome, white: RFP. Homozygous clones are RFP negative. Note that the homozygous clones are similar to the heterozygous cysts in region 1 but contain abnormally small ring canals in region 2, similar to *flw^XE55A^*.
(ZIP)
