# Supplementary figures and images for: Protein Phosphatase 1ß Limits Ring Canal Constriction during Drosophila Germline Cyst Formation
Source: PLoS One. 2013 Jul 25;8(7):e70502. doi: 10.1371/journal.pone.0070502 (PMC3723691; doi:10.1371/journal.pone.0070502)

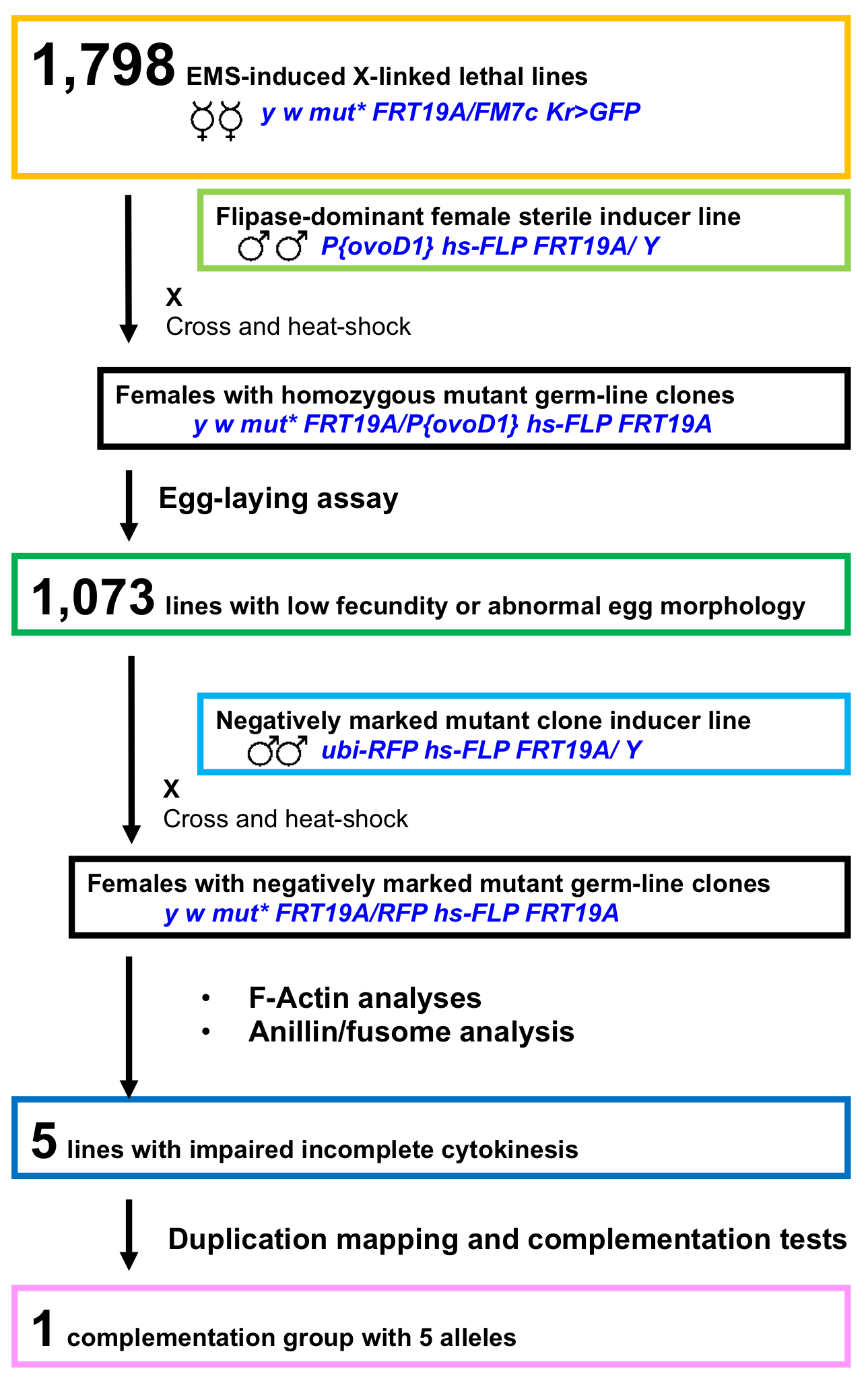

Supplement: Figure S1 — A flowchart of the procedures to isolate mutations causing defective incomplete cytokinesis. IC mutations were identified in four steps: screening the 1,798 lethal lines for mutations disrupting oogenesis, screening the 1,073 oogenesis mutations for those affecting actin ring canal morphogenesis, screening the small ring canal mutations for IC mutations, and mapping of the IC mutations. (TIF) [file pone.0070502.s001.tif]

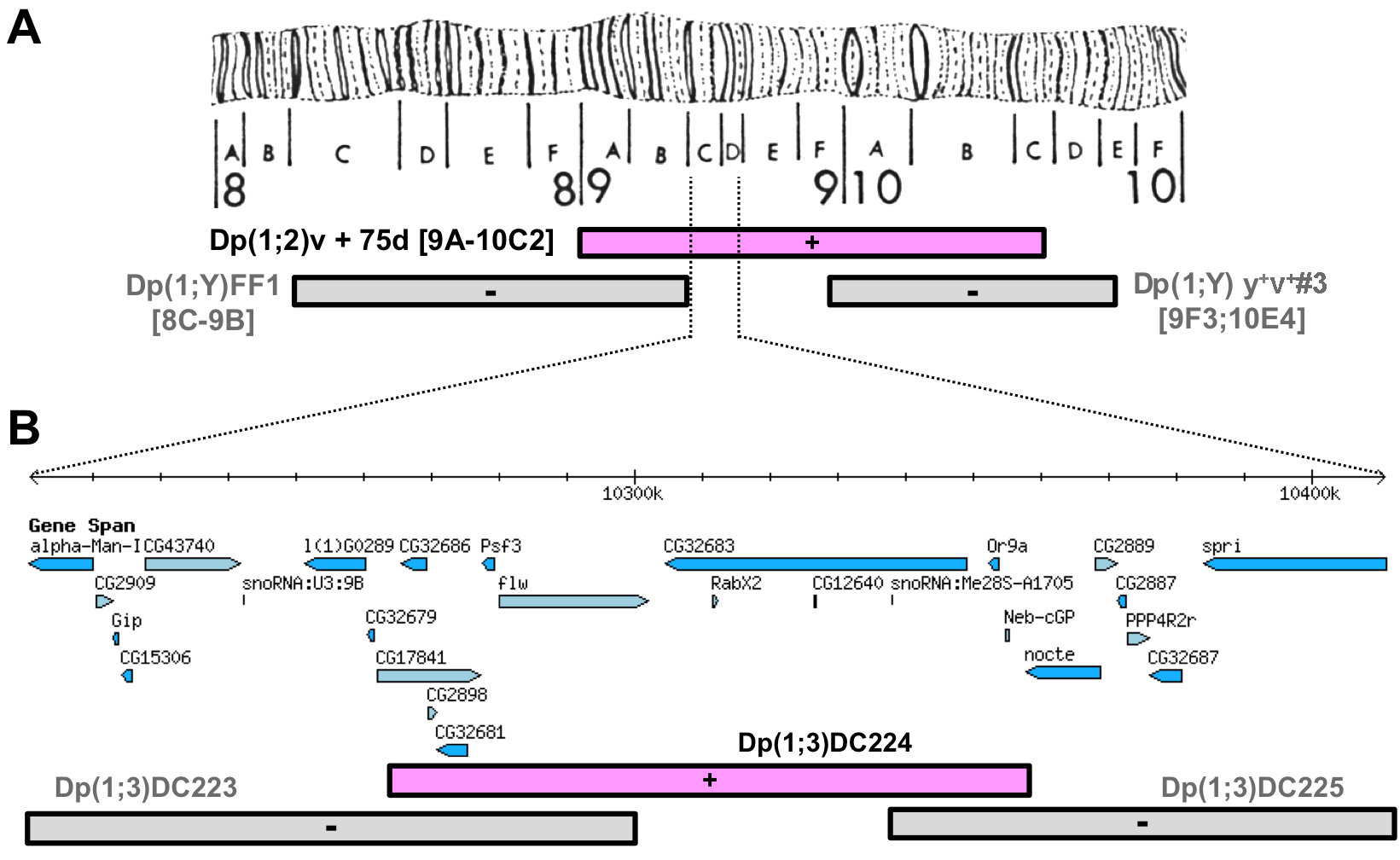

Supplement: Figure S2 — Mapping of XE55 mutations. (A) Duplication mapping of XE55s between X chromosome bands 9B1 and 9F2. Duplications were shown as boxes below a drawing (by C.B. Bridges) of part of a polytene X chromosome. The duplication that rescued the mutation is labeled in pink, while those did not rescue are in grey. Same is true for panel B. (B) Refinement of the locations of the mutations with P[acman] BAC duplications to a region containing four genes. Above the duplications is a gene annotation of the relevant chromosomal region (adapted from http://flybase.org). (TIF) [file pone.0070502.s002.tif]

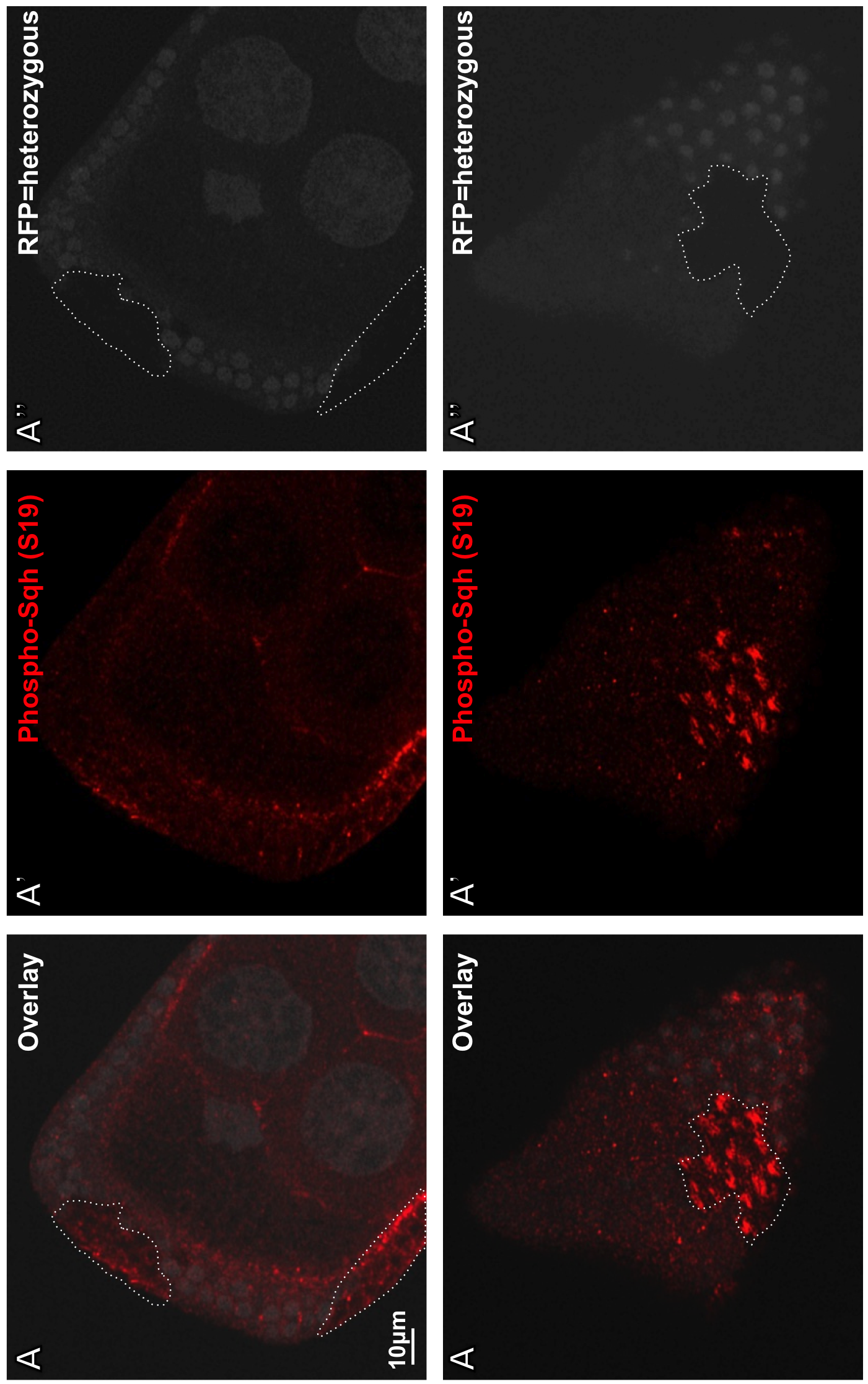

Supplement: Figure S3 — Mutations of flw in follicle cells caused an increase of phosphorylated Sqh. Immunostaining of a stage 10 egg chamber from a XE55A/ubi-RFPNLS hsFlp122 FRT19A fly with a phospho-myosin light chain 2 (Ser19) antibody (Cell Signaling Technology, Inc. #3671), which recognizes Sqh phosphorylated at Ser21 (red). Panels A and B are two different focal planes of the same egg chamber. Homozygous clones (boxed with dashed lines) were marked by the absence of RFP (white). Note the increase of phosphorylated Sqh in all the clones. Scale bar: 10 µm. (TIF) [file pone.0070502.s003.tif]
